# Successful endoscopic duodenal stent placement for afferent loop obstruction following Billroth II gastrectomy: a case report

**DOI:** 10.3389/fonc.2025.1672367

**Published:** 2025-10-15

**Authors:** Shanliang Ye, Yong Peng, Wenhang Zhuang, Zhiqiang Mo

**Affiliations:** ^1^ Department of Gastroenterology, Guangdong Provincial People’s Hospital (Guangdong Academy of Medical Sciences), Southern Medical University, Guangzhou, China; ^2^ Department of Endoscopy Center, Ganzhou Hospital of Guangdong Provincial People’s Hospital, Guangzhou, China; ^3^ Department of Minimally Invasive Intervention, Guangdong Provincial People’s Hospital (Guangdong Academy of Medical Sciences), Southern Medical University, Guangzhou, China

**Keywords:** afferent loop obstruction, Billroth II gastrectomy, endoscopic stent placement, interventional radiology, gastrointestinal obstruction

## Abstract

**Background:**

Afferent loop obstruction (ALO) is an uncommon but potentially life-threatening complication following Billroth II gastrectomy, with an estimated incidence of around 1%. It often presents with nonspecific symptoms such as postprandial vomiting and jaundice, making timely diagnosis and effective treatment crucial. Minimally invasive endoscopic techniques have emerged as promising alternatives to surgery.

**Case presentation:**

We report the case of a 63-year-old woman with a history of Billroth II gastrectomy for gastric cancer who presented with progressive jaundice, nausea, and vomiting. Imaging revealed significant duodenal wall thickening consistent with ALO. Conventional endoscopic attempts to traverse the obstructed segment failed due to severe luminal narrowing and tortuosity. Under combined endoscopic and interventional radiologic guidance, a duodenal self-expanding metal stent was successfully deployed across the stricture, resulting in immediate symptom relief and biochemical improvement.

**Conclusion:**

Interventional-guided endoscopic stent placement is a safe, effective, and minimally invasive approach for managing malignant or benign ALO in post-gastrectomy patients. This hybrid technique may be particularly valuable in anatomically complex or surgically high-risk cases.

## Introduction

Billroth II gastrectomy, a commonly employed reconstructive technique following distal gastrectomy (1, 2), involves the creation of a gastrojejunostomy with exclusion of the duodenal stump (3). While this approach effectively restores gastrointestinal continuity and facilitates postoperative recovery, the resultant alteration in anatomy can give rise to a range of unique complications. One such complication is afferent loop obstruction (ALO), a rare but potentially life-threatening condition with an estimated incidence of approximately 1% (4, 5).

ALO occurs when there is mechanical obstruction of the afferent limb—typically caused by postoperative adhesions, anastomotic strictures, kinking of the bowel, or internal herniation (6). Acute ALO usually presents soon after surgery with sudden abdominal pain, nausea, and vomiting (7), whereas chronic ALO develops months to years later with more gradual symptoms such as postprandial discomfort and recurrent bilious vomiting (8). This obstruction leads to the accumulation of bile and pancreatic secretions within the obstructed segment, manifesting clinically as postprandial abdominal pain, bilious vomiting, and, in severe cases, obstructive jaundice or cholangitis (9). Timely diagnosis is crucial, as delayed intervention may result in afferent limb perforation, sepsis, or other serious complications.

Traditionally, surgical revision was the primary treatment (10), but endoscopic duodenal stenting has gained traction as a less invasive alternative, particularly in high-risk patients (11). Herein, we present a technically challenging case of ALO successfully managed via interventional radiology-assisted endoscopic stenting, highlighting its feasibility and advantages.

## Case report

**Figure 1:**
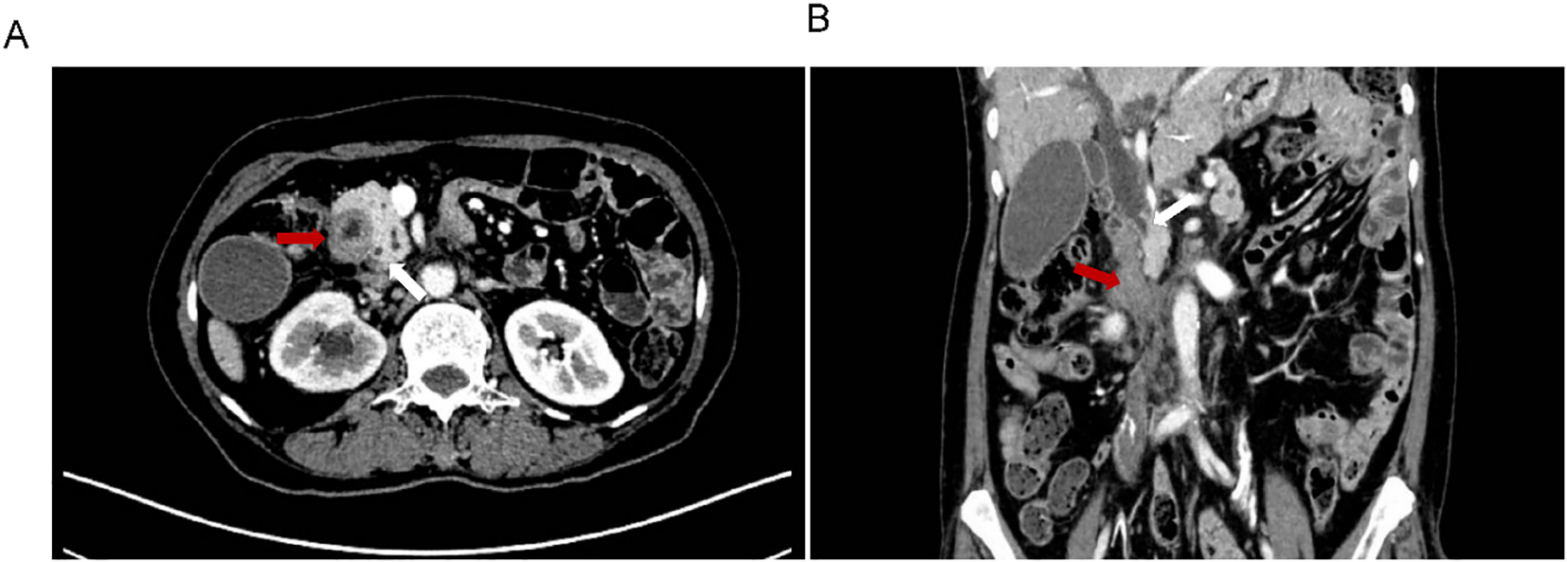
Preliminary PET-CT exam. The location of Duodenal lesions (red arrow) and bile duct obstruction (blue arrow) in coronal **(A)** and transverse **(B)** positions.

A 63-year-old woman with a prior history of Billroth II gastrectomy for gastric cancer presented in November 2024 with progressive jaundice, persistent nausea, bilious vomiting, and anorexia. Laboratory findings indicated obstructive cholestasis, and PET-CT demonstrated marked thickening of the D2, D3, and D4 portions of the duodenum. The patient was initially diagnosed with obstructive jaundice based on hyperbilirubinemia. Preliminary PET-CT demonstrated duodenal lesions and bile duct obstruction (**Figure 1**). To alleviate biliary obstruction, percutaneous transhepatic cholangiography and drainage (PTCD) with stent placement were performed. Percutaneous transhepatic cholangiography initially targeted biliary drainage for distal CBD obstruction. Although a percutaneous approach was initially chosen, it only allowed placement of a biliary stent. Dilatation of the percutaneous tract to accommodate the larger duodenal stent (requiring 6–10 Fr) (12) was not feasible, necessitating an endoscopic approach for its placement. Reflux of afferent loop contents into the bile ducts was subsequently observed, prompting placement of a duodenal stent to relieve afferent loop pressure and improve both biliary and bowel obstruction. Subsequent Esophagogastroduodenoscopy (EGD) revealed ALO; However, the ultra-slim endoscope was unable to traverse the narrowed segment, confirming a high-grade stricture.

**Figure 2:**
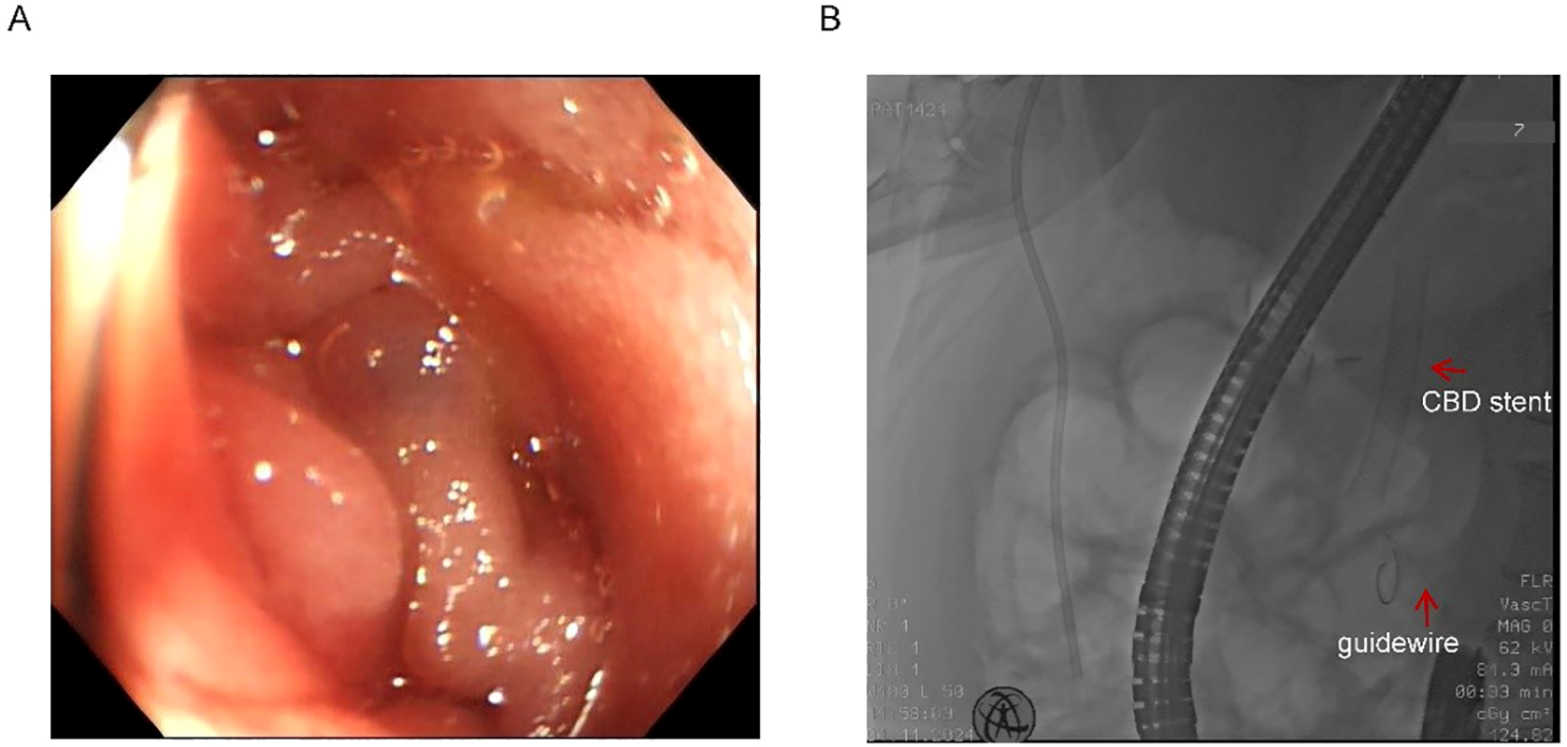
Twisted afferent loop and failed guidewire passage. **(A)** Endoscopic view of the extremely narrow and twisted afferent loop. **(B)** Failed attempts to pass the guidewire through the stricture. Red arrows indicate the previously placed CBD stent and the guidewire.

Endoscopic assessment identified a markedly narrowed, angulated afferent limb (**Figure 2A**). Multiple attempts to navigate a guidewire through the stricture via conventional endoscopy were unsuccessful due to compression and twisting of the afferent loop (**Figure 2B**). Given the patient’s severe infection and the distal location of the afferent loop obstruction, surgical intervention under general anesthesia was considered high-risk and therefore not feasible.

**Figure 3:**
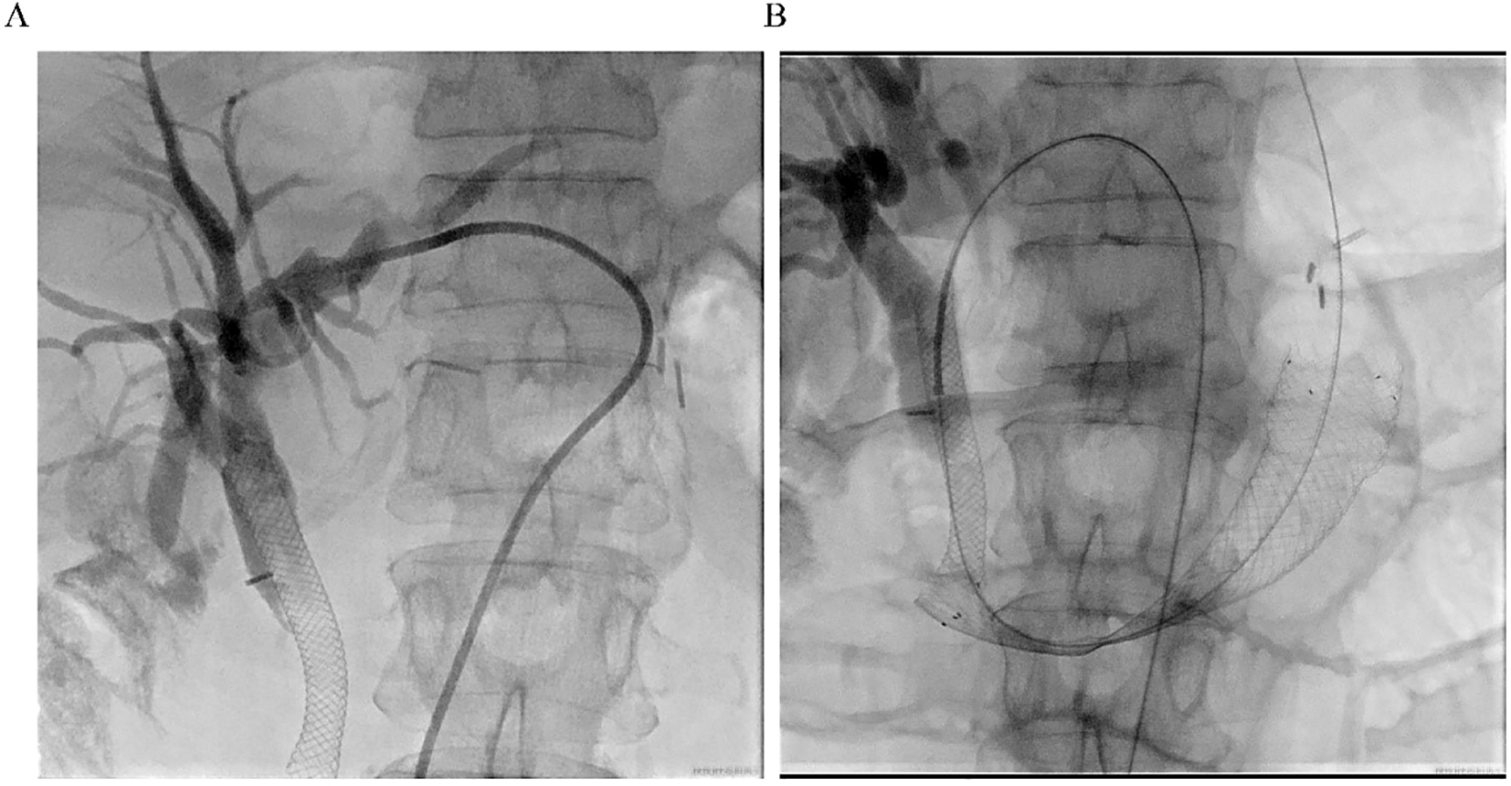
Procedural imaging. **(A)** Ultrasound-guided percutaneous transhepatic cholangiography. Intrahepatic bile duct dilatation is observed, and the contrast medium is retained above the previously placed biliary stent. **(B)** Fluoroscopic image showing the fully expanded duodenal stent in place across the high-grade stricture, with the guidewire and previously placed metal biliary stent in position.

The previous drainage catheter had been in place for an extended period and had become displaced prior to stent placement, necessitating its removal before the procedure. Under real-time ultrasound guidance, a 22G needle was used to puncture the intrahepatic bile duct via a transhepatic route, carefully avoiding the thoracic cavity, hepatic artery, and portal vein. A guidewire (0.035”) was then advanced through a catheter (5F VER 135°) directly into the distal duodenal afferent loop (**Figure 3A**). Once within the duodenum, the guidewire was manipulated proximally and successfully passed through the obstructed segment. Simultaneous EGD was performed to locate the stricture and retrieve the guidewire using a foreign body forceps. The endoscope was then retracted carefully, pulling the guidewire out through the mouth to create an internal-external access route.

A self-expanding duodenal stent (COOK EVO-22-27-9-D) was advanced over the guidewire under fluoroscopic guidance, with maintained tension on both ends to ensure stability and accurate deployment. After several positional adjustments, the stent was successfully deployed across the stricture (**Figure 3B**). Fluoroscopic imaging confirmed full expansion of the stent and restoration of luminal patency. The procedure was well tolerated, and the patient reported immediate resolution of obstructive symptoms.

**Figure 4:**
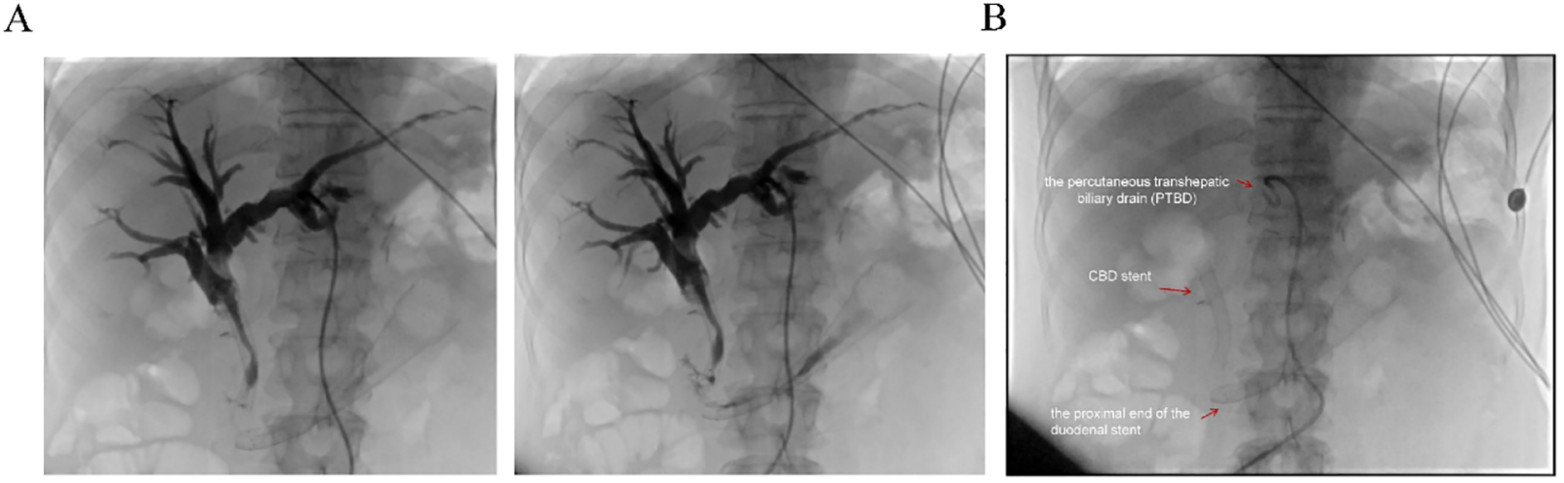
Postoperative imaging evaluation. **(A)** Post-stent placement percutaneous transhepatic cholangiography showing resolution of obstruction in both the intrahepatic bile ducts and the afferent loop. **(B)** Fluoroscopic view post-surgery demonstrating the properly positioned stent, absence of obstruction, and the patient’s subsequent clinical improvement. Red arrows indicate the proximal end of the duodenal stent, the previously placed CBD stent, and the percutaneous transhepatic biliary drain (PTBD).

Biochemical improvement was observed within four days post-procedure: total bilirubin decreased from 80.8 μmol/L to 68.3 μmol/L, direct bilirubin from 41.4 μmol/L to 33.9 μmol/L, and γ-glutamyltransferase (GGT) from 783 U/L to 377 U/L. Percutaneous transhepatic cholangiography showed resolution of obstruction in both the intrahepatic bile ducts and the afferent loop (**Figure 4A**). Follow-up EGD one week later was performed to assess stent positioning and duodenal lumen patency, and confirmed optimal stent placement with no evidence of migration or recurrent obstruction (**Figure 4B**). the patient’s clinical condition and quality of life improved significantly.

This case underscores the feasibility and therapeutic potential of interventional radiology-assisted endoscopic duodenal stent placement in the management of ALO following Billroth II reconstruction. For patients who are poor surgical candidates, this hybrid minimally invasive strategy offers a safe and effective alternative with rapid symptom resolution and favorable short-term outcomes.

## Discussion

In this case, the patient developed afferent loop obstruction (ALO) after surgery, and a combined approach of percutaneous transhepatic cholangiography and drainage (PTCD) with endoscopic duodenal stent placement achieved favorable outcomes. Considering the patient’s advanced age, postoperative infection, and anesthetic risk, this hybrid approach provided a safe and effective treatment option for high-risk patients. However, this strategy has certain limitations, including the need for multidisciplinary collaboration and high technical requirements, which may restrict its applicability in some medical centers. Future studies should evaluate the long-term outcomes of this combined approach and compare it with other treatment modalities to further validate its safety and efficacy (13).

Alternative treatment options are also worth considering, such as endoscopic ultrasound-guided gastroenterostomy (EUS-GE), which has shown favorable efficacy and safety in the management of gastric outlet obstruction (14, 15). EUS-GE may provide a minimally invasive option for patients who cannot tolerate surgery or for whom conventional endoscopic stenting has failed. However, the procedure is technically complex, requires specialized equipment, and its use in the treatment of ALO remains to be further validated.

## Data Availability

The original contributions presented in the study are included in the article/Supplementary Material. Further inquiries can be directed to the corresponding author.
